# Ecological and evolutionary dynamics to design and improve ovarian cancer treatment

**DOI:** 10.1002/ctm2.70012

**Published:** 2024-08-29

**Authors:** Grace Y. Q. Han, Monica Alexander, Julia Gattozzi, Marilyn Day, Elayna Kirsch, Narges Tafreshi, Raafat Chalar, Soraya Rahni, Gabrielle Gossner, William Burke, Mehdi Damaghi

**Affiliations:** ^1^ Renaissance School of Medicine at Stony Brook University Stony Brook New York USA; ^2^ Department of Molecular and Cellular Biology Stony Brook University Stony Brook New York USA; ^3^ Department of Molecular and Cellular Pharmacology Stony Brook University Stony Brook New York USA; ^4^ Department of Obstetrics and Gynecology Renaissance School of Medicine at Stony Brook University Stony Brook New York USA; ^5^ Cellinfinity Bio New Haven Connecticut USA; ^6^ Stony Brook Cancer Center Renaissance School of Medicine at Stony Brook University Stony Brook New York USA; ^7^ Stony Brook University Stony Brook New York USA; ^8^ Department of Obstetrics and Gynecology Stony Brook University Hospital Stony Brook New York USA; ^9^ Department of Pathology Renaissance School of Medicine at Stony Brook University Stony Brook New York USA; ^10^ Department of Radiation Oncology Renaissance School of Medicine at Stony Brook University Stony Brook New York USA

**Keywords:** ovarian cancer, phenotypic plasticity, tumour ecology, tumour evolution, tumour microenvironment

## Abstract

Ovarian cancer ecosystems are exceedingly complex, consisting of a high heterogeneity of cancer cells. Development of drugs such as poly ADP‐ribose polymerase (PARP) inhibitors, targeted therapies and immunotherapies offer more options for sequential or combined treatments. Nevertheless, mortality in metastatic ovarian cancer patients remains high because cancer cells consistently develop resistance to single and combination therapies, urging a need for treatment designs that target the evolvability of cancer cells. The evolutionary dynamics that lead to resistance emerge from the complex tumour microenvironment, the heterogeneous populations, and the individual cancer cell's plasticity. We propose that successful management of ovarian cancer requires consideration of the ecological and evolutionary dynamics of the disease. Here, we review current options and challenges in ovarian cancer treatment and discuss principles of tumour evolution. We conclude by proposing evolutionarily designed strategies for ovarian cancer, with the goal of integrating such principles with longitudinal, quantitative data to improve the treatment design and management of drug resistance.

**Key points/Highlights:**

- Tumours are ecosystems in which cancer and non‐cancer cells interact and evolve in complex and dynamic ways.
- Conventional therapies for ovarian cancer inevitably lead to the development of resistance because they fail to consider tumours’ heterogeneity and cellular plasticity.
- Eco‐evolutionarily designed therapies should consider cancer cell plasticity and patient‐specific characteristics to improve clinical outcome and prevent relapse.

## Graphical abstract

1. Tumours are ecosystems in which cancer and non‐cancer cells interact and evolve in complex and dynamic ways.

2. Conventional therapies for ovarian cancer inevitably lead to the development of resistance because they fail to consider tumours’ heterogeneity and cellular plasticity.

3. Eco‐evolutionarily designed therapies should consider cancer cell plasticity and patient‐specific characteristics to improve clinical outcome and prevent relapse.

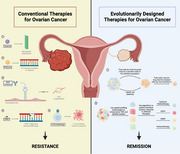

## 1 BACKGROUND

Despite many new developments in treatment, ovarian cancer remains a leading cause of cancer‐related death in women, with a 5‐year survival rate of 31% for advanced stage disease.^1^ The most commonly occurring (90%) ovarian malignant tumours are epithelial in histopathological origin, which is the focus of the review. Epithelial ovarian cancer (EOC) is itself divided into five major subtypes: high‐grade serous OC (HGSOC, 70%), low‐grade serous OC (LGSOC, <5%), endometrioid (10%), clear cell (10%) and mucinous (3%). Each subtype displays a unique profile of molecular changes, clinical behaviours and treatment outcomes. The remaining 10% of ovarian malignant tumours include germ cell tumours, sex cord‐stromal tumours and extremely rare small‐cell carcinomas.^2^, ^3^ In 2023, ovarian cancer was the fifth most deadly cancer among females in the United States, accounting for an estimated 5% of cancer‐related deaths.^1^ The high mortality is largely due to symptoms presenting in advanced disease (stage III and stage IV), leading to late diagnosis. Additionally, routine screening with ultrasound and blood testing have not been proven to reduce morbidity from the disease and are not regularly recommended.^4^ The lack of effective screening tools is also reflected economically as the average cost of ovarian cancer treatment remains the highest among all cancer types, ranging from USD 80 000 in the first year to USD 100 000 in the final year. Over the last decade, efforts have been made to search for a more cost‐effective, accessible approach to ovarian cancer prevention and improving clinical outcomes.^5^ According to the NCCN Clinical Practice Guidelines in Oncology (NCCN Guidelines), frontline treatment for ovarian cancer may include cytoreductive surgery and platinum‐ and taxane‐based chemotherapy.^6^ Furthermore, targeted therapies, such as bevacizumab (an antiangiogenic) and poly ADP‐ribose polymerase inhibitors (PARPis), have gained FDA approvals for the treatment of ovarian cancer. Despite increasing therapeutic options, the eventual development of drug resistance remains a pressing problem and a proximate cause of death in ovarian cancer.^7^

Normal ovarian cells are routinely challenged with stressors after puberty. Ovarian ageing is regulated by a variety of processes that, when dysregulated, can lead to cancer. These processes include changes in DNA methylation, decreased damage remodelling capacity, increased fibrosis, increased DNA damage and increased cytoskeletal defects.^8^ Once cancer arises, a variety of existing factors within the tissue environment dictate disease progression and cell response to therapeutic interventions. Different subregions in a tumour microenvironment (TME) evolve in response to varying constraints, reflecting the inherent plasticity of cancer cells. This heterogeneity makes each patient's disease unique, shaped by its oncogenic evolutionary history.^9^ For advanced, metastatic disease, cancer cell plasticity is necessary for the creation of new niches in distant sites with microenvironments that differ greatly from the primary tumour. Establishment of these clones further increases the heterogeneity of the disease and complicates eradication with existing therapies.

Given this complexity, it is not surprising that current approaches continue to fall short in effectively treating ovarian cancer. Instead, new strategies that more accurately and comprehensively consider the dynamic and heterogeneous eco‐evolutionary processes underlying cell populations are required to manage drug resistance and design more patient‐specific treatments.^9^ In this review, we address the gap between clinical practice and evolutionary biology of cancer research on ovarian cancer management. We provide an overview of the current state of ovarian cancer treatment, discuss key biological resistance mechanisms and highlight emerging immunotherapeutic and combination approaches. Subsequently, we discuss the ecological and evolutionary principles of ovarian cancer. To conclude, we present next‐step strategies for the integration of eco‐evolutionary‐based approaches to tackle ovarian cancer and drug resistance more effectively.

## 2 OVARIAN CANCER TREATMENTS AND MECHANISMS OF RESISTANCE

### 2.1 Treatment overview for ovarian cancer

**FIGURE 1:**
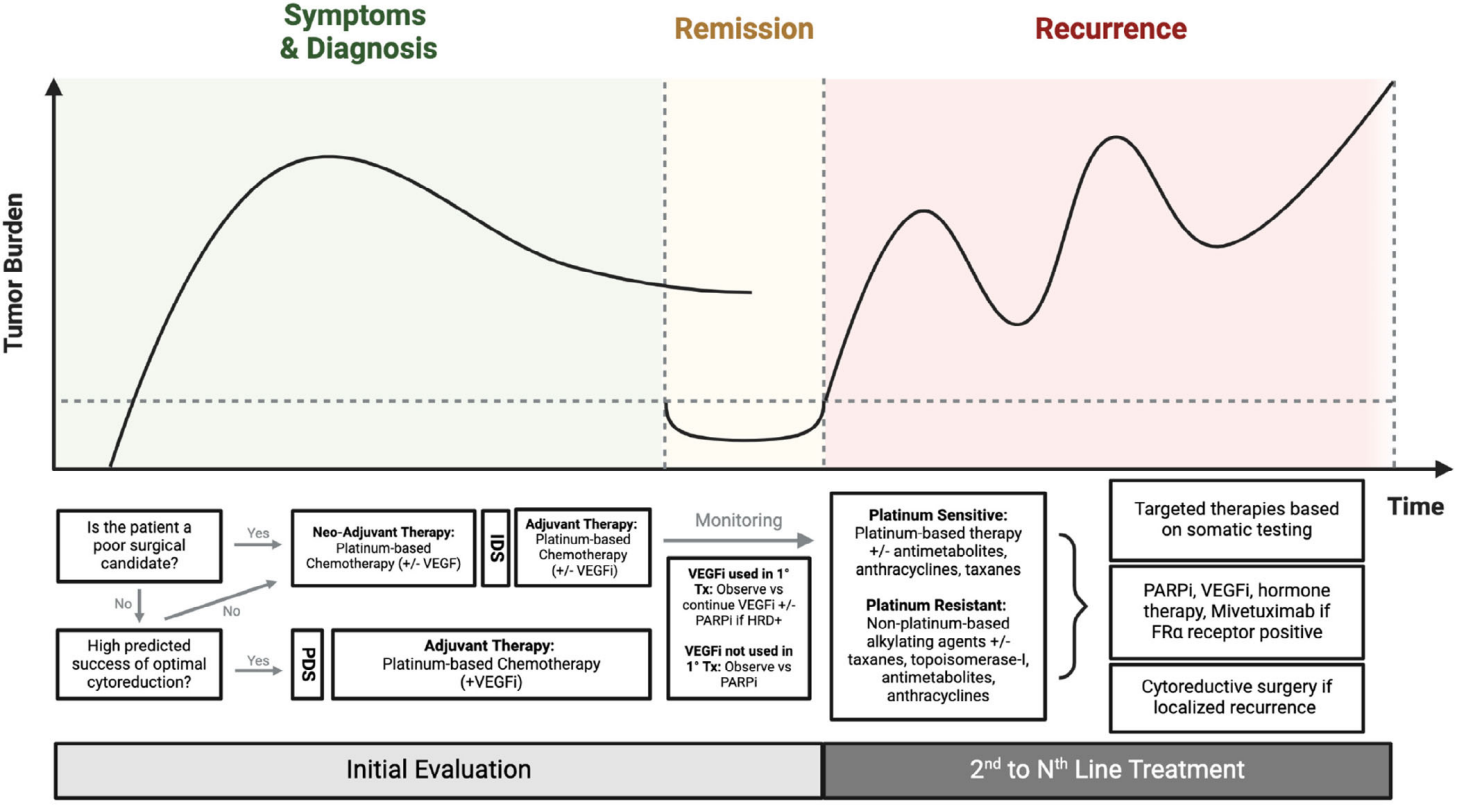
Timeline illustrating a typical clinical trajectory in the treatment of high‐grade serous ovarian cancer, the most common type of ovarian cancer. Depending on multiple factors such as tumour stage or physical condition, the patient will either undergo immediate debulking surgery after diagnosis or be first treated with neo‐adjuvant therapy. This is followed by adjuvant and maintenance therapy, if appropriate. However, while cancers often respond well initially, they have a high likelihood of developing resistance and recurrence. FR‐α, folate reductase alpha; HRD, homologous recombination deficient; IDS, interval debulking surgery; PARPi, poly ADP‐ribose polymerase inhibitors; PDS, primary debulking surgery; VEGFi, vascular endothelial growth factor inhibitors.

A combination of platinum‐ and taxane‐based chemotherapy remains the backbone of most treatment regimens for EOC. Systemic therapy, administered before (neo‐adjuvant therapy) or after surgery (adjuvant therapy), is typically recommended for advanced stage cancers, high‐grade histology and recurrent diseases.^10^ After initial treatment, patients are monitored closely and may be offered maintenance therapy, consisting of PARPis and vascular endothelial growth factor inhibitors (VEGFis), to prolong progression‐free survival (PFS). Patients who showed a partial or complete response to VEGFis during initial treatment are often continued on VEGFis for maintenance therapy. The addition of PARPis depends on the presence of a germline or somatic homologous recombination (HR) deficiency.^6^ If VEGFis were not used, PARPis can be used alone (Figure 1).

Methods for monitoring recurrence largely depend on the grade and stage of the initial cancer. Clinicians often rely on a combination of clinical examinations, imaging and biomarkers to accurately evaluate treatment response and recurrence.^10^ However, there is no single method for detecting recurrent ovarian cancer that has been shown to benefit overall survival (OS). Computed tomography (CT) is routinely used to diagnose and stage ovarian cancer patients. Positron emission tomography combined with CT (PET/CT) is the recommended imaging modality for detecting malignant lymph nodes, peritoneal metastases and recurrent disease. According to a recent meta‐analysis, magnetic resonance imaging (MRI) had a sensitivity of 91% and specificity of 85% for detecting ovarian cancer, outperforming CT and PET/CT.^11^ Despite the promising evidence, MRI is often not used clinically (at least in the United States) due to difficulty with prior authorisations and insurance coverage. Serum CA125 levels, elevated in 50% of early‐stage and 92% of advanced‐stage ovarian tumours, are used to evaluate treatment response and is a validated predictor for detecting HGSOC recurrence.^12^ Currently, CA125 and HE4, another serum biomarker, are the only approved biomarkers for EOC detection. Emerging evidence shows that a combination of the two biomarkers provides a higher specificity and sensitivity than either used alone.^13^ Over the last two decades, miRNAs have demonstrated remarkable potential in predicting ovarian cancer. miRNAs are short, single‐stranded RNA segments that are secreted and detectable in all bodily fluids, extracellular vesicles and the tissue microenvironment. However, for miRNAs to be accepted as reliable biomarkers clinically, more research is needed to standardise sample processing and optimise miRNA detection in tumours and blood.^13^

If cancer recurs, subsequent lines of chemotherapy are employed (Figure 1). The choice of agents depends on the patient's initial response and presence of resistance. Platinum‐sensitive refers to effective platinum‐based treatment where recurrence occurred more than 6 months after therapy. In this case, platinum‐based therapies are used again in combination with other agents administered during previous treatment.^6^ Conversely, platinum‐resistant cancer refers to recurrence within 6 months of platinum‐based treatment.^6^ Here, subsequent treatments include non‐platinum chemotherapy, such as alkylating agents, antimetabolites, taxanes, anthracyclines, topoisomerase inhibitors, VEGFis, PARPis and hormone therapies.

### 2.2 Cytotoxic chemotherapy

**FIGURE 2:**
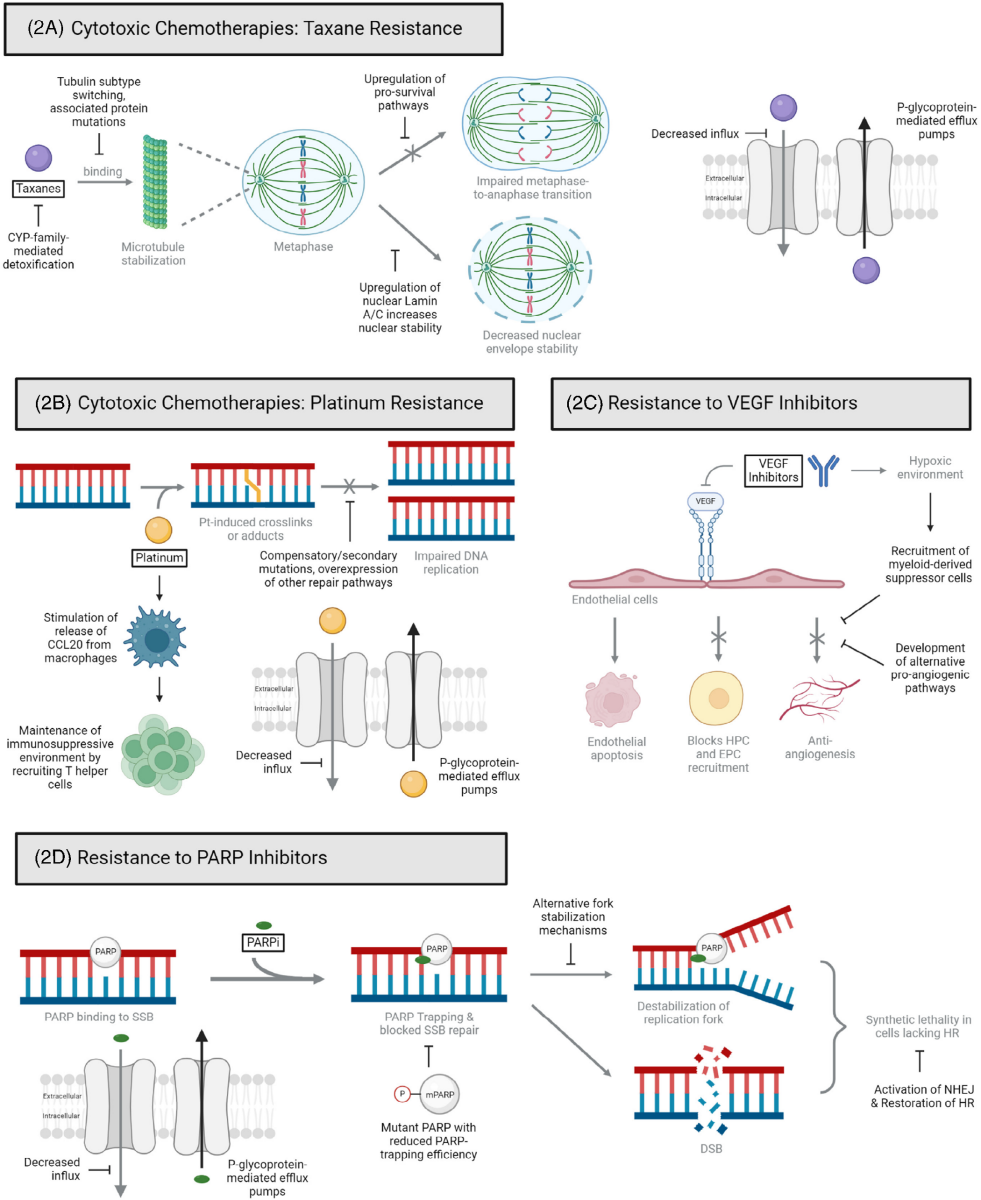
Diagrams outlining the resistance mechanisms of some commonly used systemic therapies. Cytotoxic chemotherapies such as (A) taxanes and (B) platinum‐based agents are approved for first‐line treatment. Taxanes work by inhibiting mitotic progression and destabilising the nuclear envelope. Platinum‐based agents work by introducing adducts that prevent replication. (C) Vascular endothelial growth factor (VEGF) inhibitors are anti‐angiogenic agents. (D) Poly‐ADP ribose polymerase inhibitors (PARPis) mainly induce cytotoxicity by trapping PARP at the lesion, preventing fork progression and repair to destabilise the junction. This amounts to synthetic lethality. Common resistance pathways include secondary or compensatory mutations, use of alternate pathways, upregulation of drug efflux by P‐glycoprotein pumps and changing conditions that favour tumour promotion. CYP, cytochrome P450; DSB, double‐stranded break; EPC, epithelial progenitor cell; HPC, haematopoietic stem cell; HR, homologous repair; NHEJ, non‐homologous end joining; SSB, single‐stranded break.

Taxanes and platinum‐based agents exert their cytotoxicity by impairing normal DNA and cellular replicative functions via microtubule stabilisation and generation of DNA crosslinks via alkylation, respectively^14^ (Figure 2A,b). These chemotherapies are FDA approved and recommended by NCCN for ovarian cancer in both frontline and recurrent settings.^6^

Taxane‐stabilised microtubules cause irreversible nuclear envelope rupture and micronuclei formation in ovarian cancer cells.^15^ Paclitaxel‐induced cell death is also associated with decreased expression of Lamin A/C, leading to lower nuclear membrane stability.^15^ The mechanism behind taxane resistance is multi‐faceted, including taxane‐metabolising enzymes, dysregulation of drug efflux and influx, alteration of microtubule proteins and tubulin isotypes and upregulation of pro‐survival pathways^14^ (Figure 2A). Taxane resistance can be overcome clinically by metronomic administration. A clinical trial of weekly low‐dose single‐agent paclitaxel in platinum‐ and paclitaxel‐resistant ovarian cancer demonstrated an objective response rate of 20.9%.^16^

Multiple intrinsic and acquired mechanisms of platinum resistance have been identified (Figure 2B). Intrinsic chemotherapy resistance via increased expression of multi‐drug resistance protein 2 is linked to cisplatin resistance at cancer occurrence.^17^ Conversely, extrinsic chemotherapy resistance develops in response to drug exposure. Cisplatin can stimulate the release of CCL20 from macrophages, which maintains an immunosuppressive environment while inducing the epithelial‐to‐mesenchymal transition (EMT) that enhances tumour cell invasiveness.^18^ Other proposed mechanisms include defective drug accumulation, upregulation of drug efflux P‐glycoprotein pumps (PGP), decreased membrane transport and improved tolerance to DNA damage. A known mechanism driving platinum resistance is the upregulation of DNA damage repair pathways, such as HR that includes Breast Cancer genes 1 and 2 (BRCA1 and BRCA2). Therefore, cells that are HR deficient and initially platinum sensitive may become platinum resistant by restoring wildtype HR function.^19^

Because ovarian cancer commonly seeds the peritoneal cavity, intraperitoneal chemotherapy has been successfully used to treat advanced and recurrent EOC, sometimes in combination with hyperthermia in a technique called hyperthermic intraperitoneal chemotherapy (HIPEC) to further enhance cytotoxicity. Additionally, pressurised intraperitoneal aerosol chemotherapy is being studied in phase 1 and 2 clinical trials. While there is no consensus on the utility of HIPEC in cytoreductive surgery for primary advanced ovarian cancer without neoadjuvant chemotherapy, HIPEC has been shown to provide a survival benefit when used in interval cytoreduction following neoadjuvant chemotherapy and in the recurrent setting.^20^ There may be benefits of using HIPEC in platinum‐resistant recurrent disease, with one clinical trial showing no difference in mean survival between platinum‐sensitive and ‐resistant disease when HIPEC was used, as opposed to standard treatment where platinum‐resistant disease has worsened survival.^21^

### 2.3 Vascular endothelial growth factor inhibitors

VEGF is a key mediator of angiogenesis, promoting endothelial cell proliferation, migration and survival.^22^ Given cancer cells’ uniquely high demand for oxygen and nutrients, and thus their reliance on angiogenesis for sustained malignant growth, VEGFis are often used in cancer treatment. Bevacizumab, the most well‐established anti‐angiogenic agent, is widely used in combination with cytotoxic chemotherapy for stage II–IV ovarian cancer patients in both treatment and maintenance settings.^6^

Despite the efficacy of bevacizumab, both primary and acquired resistance to the drug are of concern (Figure 2C). Since VEGFis create a hypoxic environment, resistance often arises through the development of alternative pro‐angiogenic or neovascularisation pathways.^23^ Tumours may take over existing vessels, gain endothelial cell characteristics to form ‘vascular‐esque’ structures capable of transporting blood, or recruit myeloid cells and cancer‐associated fibroblasts to assist with vascular expansion. Hypoxia‐mediated upregulation of granulocyte‐macrophage colony‐stimulating factor (GM‐CSF) expression can also recruit myeloid‐derived suppressor cells, which exert immunosuppressive effects in the TME and lead to anti‐VEGF resistance.^24^

### 2.4 Poly ADP‐ribose polymerase inhibitors

PARPs are key proteins involved in base‐excision repair. Upon sensing and binding to single‐stranded breaks (SSBs) in DNA, they synthesise ADP‐ribose polymers that recruit repair proteins to the site (Figure 2D). Many tumours, including ovarian, upregulate PARPs, causing an accumulation of mutations that further disease progression.^25^ Without PARP activity, SSBs become double‐stranded breaks (DSBs) that must be repaired through either of two pathways: high fidelity HR or error‐prone non‐homologous end joining (NHEJ). HR‐directed repair occurs in the S/G2 phase of cell cycle and relies on BRCA1/2 activity, which is often defective or absent in ovarian cancer patients. In HR‐deficient cells, PARP inhibition directs DSB repair to NHEJ, resulting in genomic instability and cell death.^26^ NHEJ is faster than HR and takes place mainly in the G1 phase but throughout all cell cycle phases except for the M phase. Several proteins involved in NHEJ have been identified, including Ku70/80, DNA‐PKcs, Artemis, DNA pol λ/μ, DNA ligase IV‐XRCC4, and XLF, PAXX, MRI/CYREN, TARDBP of TDP‐43, IFFO1, ERCC6L2, and RNase H2.^27^ In NHEJ, the Ku70/80 heterodimer binds to DSBs and recruits DNA‐PKcs. The resulting DNA‐PK complex will recruit XRCC4, XLF, and DNA ligase IV to ligate the non‐homologous DNA ends. Typically, PARP1 PARylates (synthesises and adds PARs to its substrate) Ku70/80 and DNA‐PKcs to inhibit NHEJ. However, by inhibiting PARP1, PARPis redirect DNA repair to NHEJ.^28^, ^29^

PARPis kill cancer cells by employing synthetic lethality. This is a phenomenon in which an inactivation in either of two genes individually is inconsequential, but a concurrent mutation in both is lethal. This makes BRCA1/2‐deficient tumour cells particularly sensitive to PARPis.^30^ In addition to preventing PARP from initiating SSB repair, a more significant contributor to PARPi cytotoxicity is PARP trapping. In PARP trapping, the enzyme is crosslinked at the lesion, triggering the collapse of the replication forks and subsequent cell death.^31^

Overall, PARPis have shown efficacy as maintenance therapy after response to platinum‐based chemotherapy^32^ and in the treatment of recurrent ovarian cancer, and there are currently three which are FDA approved.^33^ Olaparib, the most widely used, is approved for maintenance therapy in patients with BRCA mt advanced and recurrent ovarian cancer and as first‐line maintenance in combination with bevacizumab for HR‐deficient advanced ovarian cancer. Rucaparib is approved as maintenance therapy in platinum‐sensitive recurrent disease only. Niraparib is the only PARPi approved for use in both BRCA mutant and wildtype in ovarian cancer and is used as first‐line maintenance therapy in platinum‐sensitive disease, although the sensitivity to/survival benefit from niraparib in HR‐proficient tumours is decreased compared to HR‐deficient tumours.^34^ PARP inhibition has been instrumental in treating HGSOC, which comprises nearly 70% of all OC cases, where up to 50% of patients are considered HR deficient.^35^ Importantly, two key considerations must be noted for the use of PARPis. First, patients with somatic/genetic BRCA1/2 mutations are significantly more susceptible to PARPis.^10^ Second, platinum‐resistant tumours respond less to PARPis, and the use of PARPis is associated with development of platinum resistance.^36^

Resistance to PARPis has emerged as a clinical concern (Figure 2D). Proposed mechanisms include overexpression of PGP, loss of expression of PARP1 and 53BP1, and secondary mutations in BRCA genes.^37^ Additionally, HR capacity restoration or epigenetic changes negate the need for PARP overall, rendering an inhibitor ineffective. Other resistance mechanisms include decreased PARP trapping as a result of PARP mutations or phosphorylation, or the loss of PARP glycohydrolase, preventing PARP accumulation.^37^

### 2.5 Hormone therapy

Importantly, regulation of the ovarian environment is tightly linked to endocrine regulation. This is primarily through the action of gonadotropin‐releasing hormone (GnRH) along the hypothalamic‒pituitary‒ovary axis, which stimulates the secretion of follicle‐stimulating hormone and luteinising hormone. In turn, these gonadotropins stimulate the secretion of estrogen, progesterone and androgens by ovarian tissue to mediate ovarian tissue development and follicle maturation.^3^ Given the intimate link between ovarian function and hormone signalling, studies on the aetiology and pathogenesis of ovarian cancer have suggested ‘hormone hypotheses’ for the development of ovarian cancer. Briefly, repeated or excessive stimulation of the ovarian tissue by gonadotropins, such as during menopause or incessant ovulation in nulliparous individuals or those with poly‐cystic ovarian syndrome, may increase the risk of ovarian cancer. In contrast, high levels of progesterone, pregnancy and use of oral contraceptives may decrease the risk of ovarian cancer.^3^

Expression of estrogen and progesterone receptors has a favourable prognosis in terms of PFS and OS.^38^, ^39^ As such, several hormone therapies, including GnRH analogues, aromatase inhibitors and estrogen receptor antagonists, have been explored for the treatment of ovarian cancer.^3^ They are mostly used for treating ovarian stromal tumours and generally have a secondary role in the treatment of EOC. The exception to this is for LGSOC: the NCCN recommends that aromatase inhibitors, GnRH analogues and estrogen receptor antagonists can be used as primary therapy, alone or in combination with cytotoxic chemotherapy, for this subset of EOC.^6^ Additionally, hormone therapy has shown promising results in the maintenance of EOC, especially for LGSOC. These data are mostly from retrospective studies and small phase 2 trials, so more prospective large phase 3 clinical trials are needed.^40^

### 2.6 Immunotherapy in ovarian cancer

The ovarian TME is infiltrated by numerous immune effector cells whose presence can contribute to both tumour progression and suppression.^41^ CD8+ T and natural killer cells are largely anti‐tumour, whereas M2 macrophages support tumour growth through promoting fibroblast proliferation and extracellular matrix (ECM) deposition.^42^ Immunotherapy aims to enhance the body's anti‐tumour response by artificially harnessing these anti‐tumour cells. Significant clinical responses to immunotherapy have been seen in melanoma, lung, urothelial, and head and neck cancers. However, current immunotherapy response rates in ovarian cancer remain modest in early clinical trials.^43^ One explanation is that ovarian cancers are typically considered ‘cold’ tumours with low numbers of infiltrating immune cells, making them less susceptible to immune‐mediated destruction.^44^ In addition, its heterogeneous TME is often characterised by a lack of nutrients, the presence of immunosuppressive cells and harsh conditions such as acidity, oxidative stress and hypoxia.^45^ These factors create a hostile environment for infiltrating immune cells, limiting their survival and functional abilities. Here, we discuss adoptive cell therapy (ACT) and immune checkpoint inhibitors (ICIs), with the hopes of extending the promising results seen in other cancers to ovarian cancer.

#### 2.6.1 Adoptive cell therapy

**FIGURE 3:**
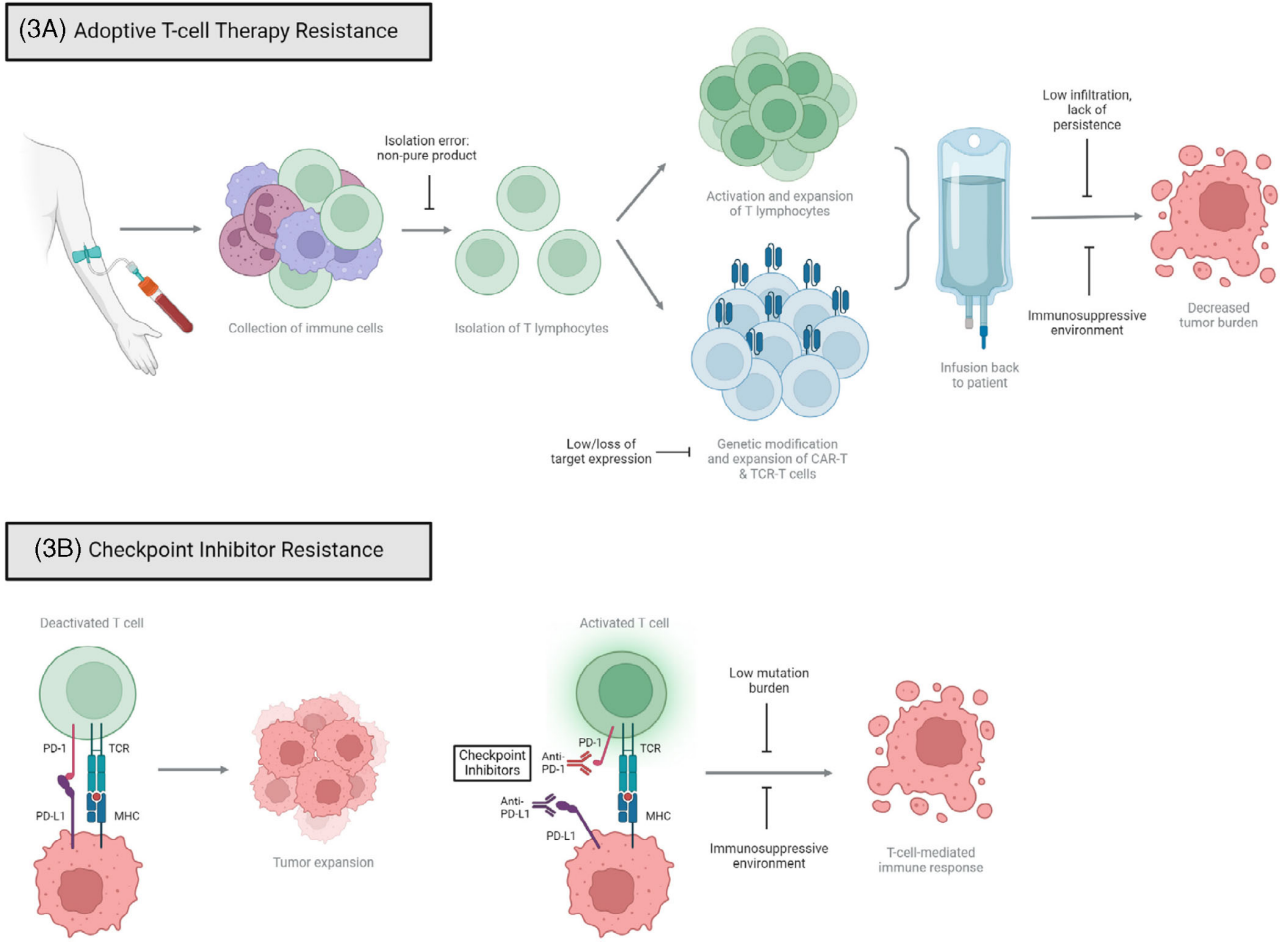
Resistance mechanisms of immunotherapies under investigation for treatment of ovarian cancer. (A) Adoptive T‐cell therapy uses a patient's modified and expanded T cells to enhance the immune response. This includes tumour‐infiltrating lymphocytes (TILs), chimeric antigen receptor T cells (CAR‐T) and T‐cell receptor T cells (TCR‐T). (B) Checkpoint inhibitors target antigen modulators on T cells and immune cells that typically lead to immune suppression. These commonly include the tumour microenvironment's (TME's) suppressive nature and antigen escape, in which the target for the therapeutic is too lowly expressed to be of use or mutation occurs. MHC, major histocompatibility complex; PD‐1, programmed cell death protein 1; PD‐L1, PD‐1 ligand; TCR, T‐cell receptor.

ACT is a promising immunological approach that employs the patient's own immune cells (autologous lymphocytes isolated from tumour or peripheral blood) to eradicate cancerous cells (Figure 3A). After they are modified and stimulated in vitro in the presence of recombinant interleukin‐2 (IL‐2) or IL‐7/IL‐15, the immune cells are infused back into the patient intravenously.^46^ To effectively eliminate tumour cells, adoptive T cells must be generated in sufficient numbers in vitro, successfully migrate and infiltrate into the tumour, overcome inhibitory networks in the TME, directly recognise tumour antigens, and persist to generate a sufficiently robust anti‐tumour response.^46^ Below, we discuss tumour‐infiltrating lymphocytes (TILs), T‐cell receptor T‐cell therapy (TCR‐T) and chimeric antigen receptor T‐cell therapy (CAR‐T). These approaches have been tested in preclinical ovarian cancer models and evaluated in early‐phase clinical trials.

TILs are endogenous autologous T cells isolated from tumour tissues, exhibiting certain tumour specificity and major histocompatibility complex restriction.^46^ They are expanded ex vivo with a high dose of recombinant IL‐2. Selected TILs with the best tumour reactivity are grown in a ‘rapid expansion protocol’ for 2 weeks and infused back into the patient. To enhance the transferred TILs’ access to the tumour sites, lymphocyte depleting chemotherapy may also be administered.^46^ In melanoma patients, adoptive immunotherapy with TILs has shown a response rate of about 50%.^47^ These approaches have been tested in preclinical ovarian cancer models and there are currently many phase 1 and 2 trials evaluating ACT in advanced and recurrent ovarian cancer (Table 1). Lifileucel (marketed as Amtagvi), a TIL therapy for treating unresectable or metastatic melanoma, recently became the first cell therapy to receive FDA approval for solid tumours.^48^

**TABLE 1:** Active and recruiting clinical trials studying adoptive T‐cell therapies in ovarian cancer.

NCT number	Title	Year started	Status
NCT03691376^121^	A phase I, open‐label study evaluating the safety and efficacy of adoptive transfer of autologous NY‐ESO‐1 CD8‐TCR engineered T cells and NY‐ESO‐1 CD4‐TCR engineered HSCs after a myeloablative conditioning regimen, with administration of IL‐2 in patients with recurrent or treatment refractory ovarian, fallopian tube or primary peritoneal cancer	2019	Active, not recruiting
NCT03017131^122^	A phase I open‐label clinical trial evaluating the safety and efficacy of adoptive transfer of NY‐ESO‐1 TCR engineered autologous t cells in combination with decitabine in patients with recurrent or treatment refractory ovarian cancer	2017	Active, not recruiting
NCT05397093^123^	A phase 1a/1b, open‐label, multicentre study evaluating the safety and feasibility of ITIL‐306 in subjects with advanced solid tumours	2022	Active, not recruiting
NCT03686124^124^	A phase 1 study evaluating genetically modified autologous T cells expressing a TCR recognising a cancer/germline antigen as monotherapy or in combination with nivolumab in patients with recurrent and/or refractory solid tumours	2019	Recruiting
NCT01174121^125^	A phase II study using short‐term cultured, autologous tumour‐infiltrating lymphocytes following a lymphodepleting regimen in metastatic cancers plus the administration of pembrolizumab	2010	Recruiting
NCT03412877^126^	A phase II study using the administration of autologous T cells genetically engineered to express TCRs reactive against neoantigens in patients with metastatic cancer	2018	Recruiting
NCT05194735^127^	A phase I/II study of autologous T cells engineered using the sleeping beauty system to express TCRs reactive against cancer‐specific mutations in subjects with solid tumours	2022	Active; not recruiting
NCT05296564^128^	A phase I/II dose escalation, safety and efficacy study of anti‐NY‐ESO‐1 TCR‐gene engineered lymphocytes given by infusion to patients with NY‐ESO‐1‐expressing metastatic cancers	2022	Recruiting
NCT02830724^129^	A phase I/II study administering peripheral blood lymphocytes transduced with a CD70‐binding chimeric antigen receptor to patients with CD70‐expressing cancers	2017	Recruiting
NCT02650986^130^	A phase I/IIa study of TGF‐β blockade in TCR‐engineered T‐cell cancer immunotherapy in patients with advanced malignancies	2017	Active; not recruiting

Abbreviations: HSC, hematopoietic stem cell; IL, interleukin; TCR, T‐cell receptor; TGF‐beta, tumor growth factor‐beta.

The presence of TILs is a clinically relevant marker that correlates with improved clinical prognosis in ovarian cancer.^49^ In a comprehensive study, Goode et al. analysed over 5500 ovarian cancer patients treated with cytoreduction and standard treatment. Patients with high CD8+ TILs had nearly double the median survival compared to those with low CD8+ TILs (5.1 vs. 2.8 years, respectively).^50^ TILs are present in more than 80% of HGSOC and 70% of endometrioid tumours, while clear cell and mucinous ovarian tumours exhibit CD8+ TILs in only 51%−52% of cases.^43^

CAR‐T cells are genetically engineered T cells designed to target tumour‐specific antigens, thereby killing tumour cells (Figure 3A). They are produced by combining the tumour antigen recognition domain from a monoclonal antibody with T‐cell intracellular signalling domains.^51^ CAR‐T cell therapy has found many approved applications in hematological cancers, yet its potential in controlling and eliminating resistant, metastatic or recurrent solid cancers needs further exploration. Numerous challenges exist, including the acquisition of sufficient cells, successful T‐cell transduction, and obtaining a pure product.^52^ Solid tumours harbour a heterogenous and unique metabolic environment, along with a protective extracellular stroma that hinders penetration of the T cells to the tumour site. The production of chemokines and cytokines by solid tumours can also interfere with T‐cell binding. It has been demonstrated that intraperitoneal administration of CAR‐T cells in patients with peritoneal carcinomatosis was superior to systemic administration, resulting in better CAR‐T cell infiltration, increased tumour reduction and decreased systemic adverse effects. Similar effects were also observed in murine ovarian cancer models.^53^ In ovarian cancer, some of the most widely studied CARs target Mucin‐16 (MUC16), folate receptor‐α (FR‐α) and mesothelin.^54^ Several phase 1 and 2 clinical trials investigating the use of CAR‐T cell therapy for ovarian cancer are ongoing.^54^

Lastly, TCR‐T cells express a genetically engineered TCR alpha and beta chain pair that recognises tumour‐specific antigens^55^ (Figure 3A). Currently, preclinical studies are investigating TCR target antigens expressed in ovarian tissues, namely MAGE‐A4, WT1 and NY‐ESO‐1.^56^ However, the immunosuppressive TME can hinder the effectiveness of TCR‐T and other ACTs. Importantly, toxicity is another major issue for therapeutic administration and success. When expressed in normal tissues, the CAR and TCR target antigens can cause an immune mediated rejection known as an ‘on‐target, off‐tumour’ response. This can potentially cause damage to vital organs like the liver and lungs.^55^ In addition, cytokine release syndrome and neurotoxicity may occur, which are potentially life threatening.

#### 2.6.2 Immune checkpoint inhibitors

Immune checkpoints are inhibitory pathways comprising a series of receptor–ligand interactions that control the equilibrium between the immune response and self‐tolerance in normal cells. As a resistance mechanism, tumour cells exploit these pathways to evade tumour‐specific T‐cell killing (Figure 3B). The most extensively studied targets of the immune checkpoint pathway are programmed cell death receptor 1 (PD‐1), its ligand (PD‐L1) and cytotoxic T‐lymphocyte‐associated protein 4 (CTLA‐4). ICIs have become established treatment options in many malignancies, including advanced lung cancer and malignant melanoma.^57^

Efficacy data from clinical trials suggest that ovarian cancer responds poorly to anti PD‐1/PD‐L1 or anti‐CTLA monotherapy.^57^ Despite the high PD‐L1 expression in ovarian cancer, not all PD‐L1‐positive cases respond to treatment. This discrepancy may be explained by the low tumour mutational burden in ovarian cancer, defined as the total number of mutations. The accumulation of somatic mutations leads to expression of neoantigens, activating the immune response.^58^ Without sufficient tumour antigens, ovarian cancer often has an inadequate T‐cell response and thus lower ICI efficacy. In contrast, melanoma and non‐small‐cell lung cancer, which have a high tumour mutational burden, have demonstrated the most robust responses to PD‐1/PD‐L1 blockade.^59^

Instead, there is an increasing effort to investigate therapeutic strategies that combine ICIs with other drugs. Of note, LB‐100 is a protein phosphatase 2A (PP2A) inhibitor that promotes the production of neoantigens and cytokines and enhances T‐cell proliferation.^60^ By stimulating the T‐cell response in ovarian cancer, LB‐100 has the potential to significantly improve outcomes for patients undergoing immunotherapies. Also, a medical records review showed that PP2A mutations correlated with exceptional survival in ovarian clear cell carcinomas treated with ICIs.^61^ An ongoing clinical trial (NCT06065462) is investigating the combination of dostarlimab, a monoclonal antibody against PD‐1, and LB‐100 for the treatment of ovarian clear cell carcinoma.^62^

Combinations of ICIs with PARPis have also been explored due to the direct crosstalk between PARP‐1 and PD‐L1/PD‐1 signalling pathways.^63^ Inhibition of PARP‐1 leads to the activation of cGAS/STING/TBK1/IRF3 pathway that results in the production of pro‐inflammatory cytokines and type I interferon response, which increases the anti‐tumour immune response. Meanwhile, PARP‐1 inhibition also increases PD‐L1 expression through deactivating GSK3. As a result, the PARPi‐mediated increase in PD‐L1 has been proposed as a promising strategy that sensitises tumour cells to anti‐PD‐L1 therapy.^63^ Preliminary results from NCT 03737643 showed that paclitaxel/carboplatin + bevacizumab + durvalumab, a monoclonal antibody against PD‐L1, followed by bevacizumab + durvalumab + olaparib for maintenance significantly improved PFS in advanced ovarian cancer patients without BRCA1/2 mutation compared to treatment regimen without durvalumab and olaparib.^64^

#### 2.6.3 Possible mechanisms of resistance to immunotherapy

Immunotherapy resistance can arise from a combination of tumour‐intrinsic factors and the TME (Figure 3). In solid tumours, an immunosuppressive microenvironment significantly hinders immunotherapeutic efficacy by causing T cell exhaustion and impaired cytotoxicity.^65^ Tumour‐intrinsic factors may be categorised into two types: relapse with negative target antigen expression and relapse with positive target antigen expression. Target antigen‐negative relapse occurs due to pre‐existing tumour cells with diminished or absent target expression.^66^ Conversely, target antigen‐positive relapse occurs despite the target expression, mainly due to tumour mutations. For example, PTEN deficiency leads to reduced tumour infiltration and decreased cytotoxicity of T cells, resulting in a poor response of melanoma cells to PD‐1 immunotherapy.^67^

### 2.7 Combination therapy

Combination therapy involves using multiple drugs to target a pathway. It can more effectively depress that pathway or block compensatory responses, thereby increasing the treatment's cytotoxicity. Additionally, abrogation of one target with one drug can increase the sensitivity of a second target to another drug, leading to a synergistic effect.^68^ Recent clinical trials have explored different combinations of immunotherapy, chemotherapy, PARPis and antiangiogenic agents to improve response rates and limit tumour‐mediated immune suppression.^57^ However, it remains unclear which ovarian cancer treatment combinations are the most effective.^57^

#### 2.7.1 Maintenance regimens

The current standard treatment for primary ovarian cancer and maintenance consists of a combination of approved taxanes, carboplatins, PARPis and anti‐VEGFs (Figure 1). The combined use of bevacizumab with PARPis is a commonly used maintenance regimen associated with significantly improved PFS in patients with both wildtype and mutant BRCA status.^69^ In addition, several ongoing trials are evaluating the use of cytotoxic chemotherapy and bevacizumab in combination with other targeted agents, including pembrolizumab and olaparib (NCT 03740165),^70^ durvalumab and olaparib (NCT 03737643),^71^ anetumab ravstansine (NCT 03587311)^72^ and atezolizumab (NCT 03353831).^73^ These studies explore the potential of anti‐VEGF therapy to enhance or synergise with other targted therapiesrather than to overcome bevacizumab resistance.

#### 2.7.2 Anti‐DNA damage response regimens

The DNA damage response (DDR) is another critical target for selectively targeting cancer cells due to their high aberrant DDR processes.^68^ Ongoing clinical trials for ovarian cancer therapies are exploring DDR pathway inhibitors in combination with other approved or developing agents. For example, the PI3K pathway is frequently dysregulated in EOC, and it is often linked to chemotherapy resistance in HGSOC. PI3K inhibition not only leads to genomic instability and mitotic catastrophe but also increased replication stress and subsequent DNA damage.^74^ ATR kinase, a downstream effector of the PI3K pathway, plays a key role in maintaining DNA replication in cancer cells despite the accumulation of stalled replication forks, thus making ATR inhibition a strong anti‐cancer strategy. ATR inhibition has been shown to synergise in vitro and in vivo with drugs such as PARPi and cisplatin that interfere with DNA replication.^68^ Notably, preclinical studies indicate that combining PARPi with ATR inhibition can overcome PARPi and platinum resistance in BRCA mutant tumours.^75^

Even though PARPis are approved for use in both BRCA mutant and wildtype patients, the efficacy of PARPis varies significantly between HR‐deficient and HR‐proficient tumours, in accordance with synthetic lethality. BRCA wildtype patients with other defects in the HR pathway, such as tumour suppressor genes ATM ATR, PALB2, CHEk2, BARD1 and RAD51, also confer sensitivity to PARPis.^76^ Several preclinical studies on HR‐proficient tumours evaluated the efficacy of compounds that inhibit DDR, including PI3K, AKT, mTOR, WEE1, MEK and CDK4/6 inhibitors.^77^ Targeting these pathways has the potential to pharmacologically induce an HR‐deficient phenotype that might expand the use of PARPis to HR‐proficient cancers.

#### 2.7.3 Oncolytic virotherapy

An emerging anti‐cancer approach that targets both the cancer cells and the TME is oncolytic virotherapy. One example is the vaccinia virus (VV), an engineered DNA virus with broad cancer‐targeting capabilities and relatively high immunogenicity.^78^ It selectively infects, replicates and lyses tumour cells while sparing normal ones. Following lysis, the virus infects neighbouring cells to propagate the infection, leading to additional cell death.^78^ Contents released from the lysed cancer cells trigger an innate immune response, leading to further tumour destruction. This therapy has the potential to transform ‘cold’ tumours, which have evaded immune response, to ‘hot’ environments, thereby modulating previously immunosuppressive TMEs.^79^ Olvimulogene nanivacirepvec (Olvi‐vec), an emerging oncolytic VV, is currently undergoing a phase III clinical trial (NCT05281471) in patients with non‐resectable platinum‐resistant/refractory ovarian cancer testing the efficacy of Olvi‐vec followed by platinum‐doublet and bevacizumab therapy for resensitisation to chemotherapy.^80^ Given the promising results from phases 1 and 2, the combination of Olvi‐vec and bevacizumab has received FDA fast track designation, expediting regulatory approval.^81^

#### 2.7.4 Antibody drug conjugates

One approach for developing drugs with minimal off‐target effects is linking chemotherapeutic agents to antibodies that bind tumour‐specific ligands. FR‐α has been identified as a promising target due to its upregulation in ovarian cancer.^82^ The antibody drug conjugate Mirvetuximab soravtansine contains an FR‐α antibody conjugated to the antimitotic agent maytansinoid DM4. It received FDA approval in 2022 for the treatment of FR‐α positive, platinum‐resistant epithelial ovarian, fallopian tube or primary peritoneal cancer patients.^83^ A preclinical study demonstrated that Mirvetuximab soravtansine, when combined with carboplatin, doxorubicin or bevacizumab, demonstrated significant anti‐tumour activity in EOC models.^84^ Clinical trial NCT02606305 was completed in 2023 and demonstrated promising results of Mirvetuximab soravtansine in combination with bevacizumab in patients with FR‐α expressing platinum‐resistant ovarian cancer. The combination treatment was efficacious across all FR‐α expression levels but further improved with higher FR‐α expression.^85^

## 3 PRINCIPLES OF TUMOUR EVOLUTION AND ECOLOGY

### 3.1 Cancer cell plasticity and evolution of drug‐resistant phenotypes

Conventional approaches for understanding tumour evolution mostly assume that cancer evolution follows ‘Darwinian’ principles with a main focus on genetic and mutation fixation in the population. The primary hypothesis is that tumour cells accumulate genetic mutations over time and different stages of neoplasia, granting the cells a survival advantage.^86^ These genetic alterations also allow the affected cells to evade the drug's activity, driving therapeutic resistance. However, emerging evidence of non‐genetic or ‘Lamarckian’ processes, which entail inheritable adaptive responses to environmental stimuli complement this genetic‐centric view.^87^ For example, drug resistance can arise from drug‐tolerant persister cells that undergo gradual, inheritable phenotypic switches.^88^ This switch, from a drug‐sensitive state to a resistant one, can be facilitated by mechanisms such as lineage switching, enhancer switching, epigenetic plasticity and the reversion to more primitive cell types that maintain proliferation and survival capabilities.^86^ These dynamic changes enable cancer cells to transition between different states, endowing them with migratory, invasive and stem‐like properties. These dynamic changes enable cancer cells to transition between different states, endowing them with migratory, invasive and stem‐like properties.

The emergence of cancer stem cells (CSCs), characterised by high resistance to conventional therapies, self‐renewal capabilities and tumour‐repopulating potential, is a significant consequence of this plasticity. It is hypothesised that post‐treatment residual cells often dedifferentiate to acquire a CSC phenotype as a resistance strategy.^86^ Alternatively, multipotent cells might dedifferentiate in response to negative selection against a targeted pathway and transdifferentiate to an unaffected pathway, leading to relapse and resistance to initial therapies. These phenomena underscore the importance of plasticity in cancer evolution, exemplified in ovarian cancer where both EMT and CSC phenotype development are identified as non‐genetic sources of tumour progression.^89^

### 3.2 Methods to study and model tumour ecosystem and evolution

Tumour evolution is frequently investigated using multi‐sample sequencing, which includes multi‐region (within a single tumour), multi‐tumour (between a patient's paired primary tumours, metastases or relapses) and longitudinal sampling.^90^ Phylogenetic analyses, including identifying shared versus private mutations between sites, along with multi‐omics are also common methods to measure evolution. Even though longitudinal sampling is historically considered the most accurate method for studying clonal dynamics, it is invasive and challenging for repeated use in patients.^90^ These experimental models are also limited in the degree of ecological complexity captured as they overlook the TME.

To address these limitations, emerging techniques like patient‐derived xenografts (PDXs) and patient‐derived organoids (PDOs) have been developed. PDX models, where human tumour tissues are implanted into immunocompromised mice, retain more clinical heterogeneity and microenvironmental interactions compared to cell‐line xenograft models. However, these are constrained by the mouse‐specific environmental response, potential divergence of tumour heterogeneity, high costs, lengthy experimental timelines and limited tissue availability.^89^ With increasing evidence that tumour‐stromal cell interactions contribute to tumour survival,^91^ more representative, three‐dimensional (3D) models of TMEs are needed.

Alternatively, PDOs offer a more relevant approach to modelling cancer and investigating therapeutics. PDO cultures are established from patient ovarian cancer biopsies in a 3D synthetic basement membrane such as matrigel, along with necessary growth factors and stromal cells that would be present in the tumour. The 3D culture enables PDOs to efficiently recapitulate the architectural, histologic and genetic features of the parental environment.^89^ Traditional methods like passaging tumours as cell lines or establishing them in mouse models subjects tumours to selective pressures that may introduce genetic changes or eliminate existing diversity. PDOs minimise these pressures by preserving the tumour's original characteristics.^90^ Additionally, the establishment and maintenance of PDOs is less resource intensive than PDXs.^89^ Because PDOs can be continuously passaged, they undergo clonal expansion to accumulate variants just as they might in vivo and can therefore be sampled for multi‐omic sequencing at several time points.^90^ Importantly, samples can be imaged longitudinally and analysed with high‐resolution single‐cell sequencing methods. Three‐dimensional in vitro methods recapitulate the tumour ecosystem more realistically and therefore have become the gold standard for representing tumour heterogeneity in a laboratory setting.^89^ Furthermore, during the initial biopsy collection, a sample can be obtained without adding additional burden to the patient. This sample can be divided into two parts: one portion can be fixed and preserved for pathological examination, while the other, smaller portion can be utilised for the development of organoids. This dual approach maximises the diagnostic and research potential of the biopsy, providing comprehensive insights into the patient's condition through traditional pathology and advanced organoid modelling.^92^

Nonetheless, organoid cultures have limitations, as they self‐organise and are highly variable due to the lack of an endogenous ECM that organises the cells’ spatial distribution. With growing evidence that the ECM influences cancer progression and might even impede drug delivery,^93^ ECM populations need to be incorporated into drug models. To address this limitation, the use of additive manufacturing has been integrated into cell culture as ‘3D bioprinting’.^91^ Once factors such as bio‐ink sources are optimised, 3D bioprinting may provide a more automated and consistent way to create organoid models. Several 3D bioprinted models for various cancers, including ovarian, have already been developed.^91^ In addition, advances in multiscale analyses and imaging that observe morphological and motional dynamics further assist in organoid use and modelling. For example, light sheet microscopy has been employed to identify single‐cell and single‐organoid characteristics.^94^ When coupled with bright‐field methods, this approach allows for further characterisation, including multi‐organoid dynamic quantification. These pipelines have successfully captured and revealed complex, heterogeneous growth patterns, supported by mathematical modelling, in both murine‐ and human‐derived organoids.^94^

To incorporate non‐genetic adaptation into multi‐sample sequencing, higher‐resolution tools must continue to be integrated into existing methods. Serial sampling with bulk sequencing can misrepresent clonal ‘demographics’ by capturing only the dominant clones at each stage. Without resolution of the non‐dominant clones, this oversimplifies the population's trajectory. It suggests a potentially misleading linear genetic evolution by revealing only *accumulated* changes over time, rather than ‘snapshots’ of all clones present *throughout*.^95^ High‐resolution single‐cell RNA sequencing (scRNA‐seq) of intra‐tumoural and circulating tumour cells should be used to gain genomic, transcriptomic and epigenetic information. When applied to longitudinal and multi‐regional sampling, this high‐resolution approach can document transitions or expansions that occur at the single‐cell level before, during and after treatment. This may reveal that resistance‐causing mutations do not occur in isolation; instead, all clones in the cancer's natural history pre‐exist to varying degrees of dominance. Therapeutic treatment might favour the transcriptional or metabolic adaptation of these pre‐existing, plastic subclones, highlighting the importance of non‐genetic mechanisms in drug resistance and tumour evolution.^95^ Longitudinal sampling analyses of PDOs may include whole‐genome sequencing, scRNA‐seq, single‐cell assay for transposase‐accessible chromatin sequencing (scATAC‐seq), lineage tracing, and fluorescent imaging and immunostaining to track genetic or clonal events occurring during tumour evolution.^90^

### 3.3 Applications of eco‐evolutionary dynamics approaches and techniques in cancer research

The use of increasingly accessible rapid sequencing technologies, illustrated by recent ‘clone tracing and clonal dynamics’ studies, is key for integrating eco‐evolutionary dynamics into oncology research. Martins et al. demonstrated that driver genes of HGSOC, such as *MYC*, *PICK3CA* and *KRAS*, are overexpressed due to somatic‐chromosomal number alterations via chromosomal instability.^87^ The primary malignant ‘clonal drivers’ were identified using multi‐regional sampling to track the cancer's evolution. Importantly, the study showed correlation between the driver gene copy number and drug response. For example, spheroid samples with *MYC* amplification were more sensitive to m‐TORC1/2 inhibition than those without, while increased *KRAS* copy number showed positive correlation with AKT inhibition.^87^ These results highlight the clinical importance of understanding clonal dynamics to identify genes important for cancer survival.

Fernandez‐Mateos et al. further characterised subclones in their investigation of drug resistance and plasticity in metastatic colorectal cancer PDOs. They proposed a model in which a clone's cellular memory is determined not only by somatic genetic mutations, but also by heritable chromatin accessibility profiles, thereby defining its degree of fitness in each environment. The clone exists as a set of transcriptional programs that can produce many transcriptional phenotypes according to environmental conditions. Given the persistent heterogeneity and plasticity seen across subclones and throughout expansion, phenotypic Darwinian selection most likely occurs later in expansion.^96^

Dose‐escalation experiments have suggested that acquisition of resistance is a gradual, adaptive process accompanied by progressive transcriptional and epigenetic reprogramming in response to environmental conditions. França et al. demonstrated that BRCA‐2‐deficient HGSOC cells continuously treated with increasing doses of olaparib significantly increased their IC50, becoming resistant. Alternatively, cells that underwent rounds of treatment followed by a recovery phase reverted to a sensitive state and were deemed drug‐tolerant persisters. scRNA‐seq analyses were conducted to assess single‐cell level transcriptional profiles for the resistant lines.^88^ Each line of treatment revealed distinct subpopulations clustered across lines into states according to differentially expressed gene (DEG) profiles. Though some genes were not exclusive to grouping, the states built from each other in a series of adaptive transitions. Functional annotation of increasingly adapted states revealed metabolic and translational reprogramming under stress, as well as enrichment for genes involved in EMT. A comparison of DEGs showed that resistance markers were more highly expressed in the resistant cells relative to persisters. This suggests early partial reprogramming for increased stress tolerance upon receiving the first drug treatment, which may prime the subsequent adaptive path.^88^

## 4 NEXT STEPS—INTEGRATING ECO‐EVOLUTIONARY DYNAMICS INTO OVARIAN CANCER RESEARCH AND TREATMENT

While in vitro studies aim to uncover the mechanisms of disease progression, ultimately these findings should improve clinical practices. Currently, reliable methods to track ovarian cancer progression are limited. CA125 is not always indicative of recurrent disease and is increasingly coupled with additional imaging and clinical exams. Fludeoxyglucose F18 (FDG)/PET/CT scans have emerged as a particularly advantageous tool. These scans detect glucose metabolism, an indicator of the Warburg effect often associated with advanced and metastatic stages of cancer. We have recently shown that adaptation to harsh microenvironmental conditions in early cancer stages selects for the Warburg phenotype.^97^ In addition to tracking disease progression, another critical aspect of ovarian cancer management is determining the most appropriate surgical approach. Currently, there are no standards for identifying which patients are candidates for primary debulking surgery versus neoadjuvant chemotherapy with interval debulking surgery. However, the Fagotti scoring system has been proposed to guide providers in decision making. This model calculates a predictive index value based on tumour distribution seen on diagnostic laparoscopy. A score greater than eight has little to no chance of complete surgical resection with primary debulking surgery.^98^ Changes in the Fagotti score after neoadjuvant treatment correlates strongly with resection status, PFS and OS.^99^ Hence, incorporating FDG/PET/CT scans with the Fagotti scoring system and CA125 monitoring can provide a more comprehensive approach to managing ovarian cancer. These tools should be implemented early to identify resistant disease or aggressive nodes, select the optimal course of treatment, and monitor for effective management.

Current clinical practices are limited in their ability to treat heterogeneous ovarian cancers. They often fail to eliminate cell populations that do not respond to therapy or have the capacity to evolve and escape treatment. These populations, known as minimally residual disease, consist of cancer cells that survive initial treatments and later expand, leading to relapse.^95^ This is particularly concerning because they often fail to respond to previously used therapies. Over a decade ago, cancer therapy designs were based on oncogene addiction, an idea where tumours rely on key oncogenes for survival. This underlies the development of therapies pharmacologically targeting such pathways to reduce tumour growth.^100^ Despite their initial success, tumour cells develop a range of resistance mechanisms to evade response. Also, non‐genetic forms of tumour evolution such as epigenetic changes and phenotypic plasticity further contribute to aggressive tumour phenotypes. Given this complexity, pharmacologically targeting oncogene addictions as a sole strategy may not be the most effective. To better prevent relapse and achieve more durable responses, it is crucial to develop therapies that affect multiple drivers of tumourigenesis, including both genetic and non‐genetic factors.

The development of treatment resistance should be expected for any evolvable tumour. Thus, eco‐evolutionary principles, which consider the dynamic interactions between cancer cells and their microenvironment, need to be considered in treatment designs. Current research methods should prioritise eco‐evolutionary‐based approaches and more comprehensive models of the TME. Here, we summarise evolutionarily designed treatment options for ovarian cancer by taking advantage of the phenotypic plasticity of cancer cells to maximise treatment efficiency and prevent relapse.

**FIGURE 4:**
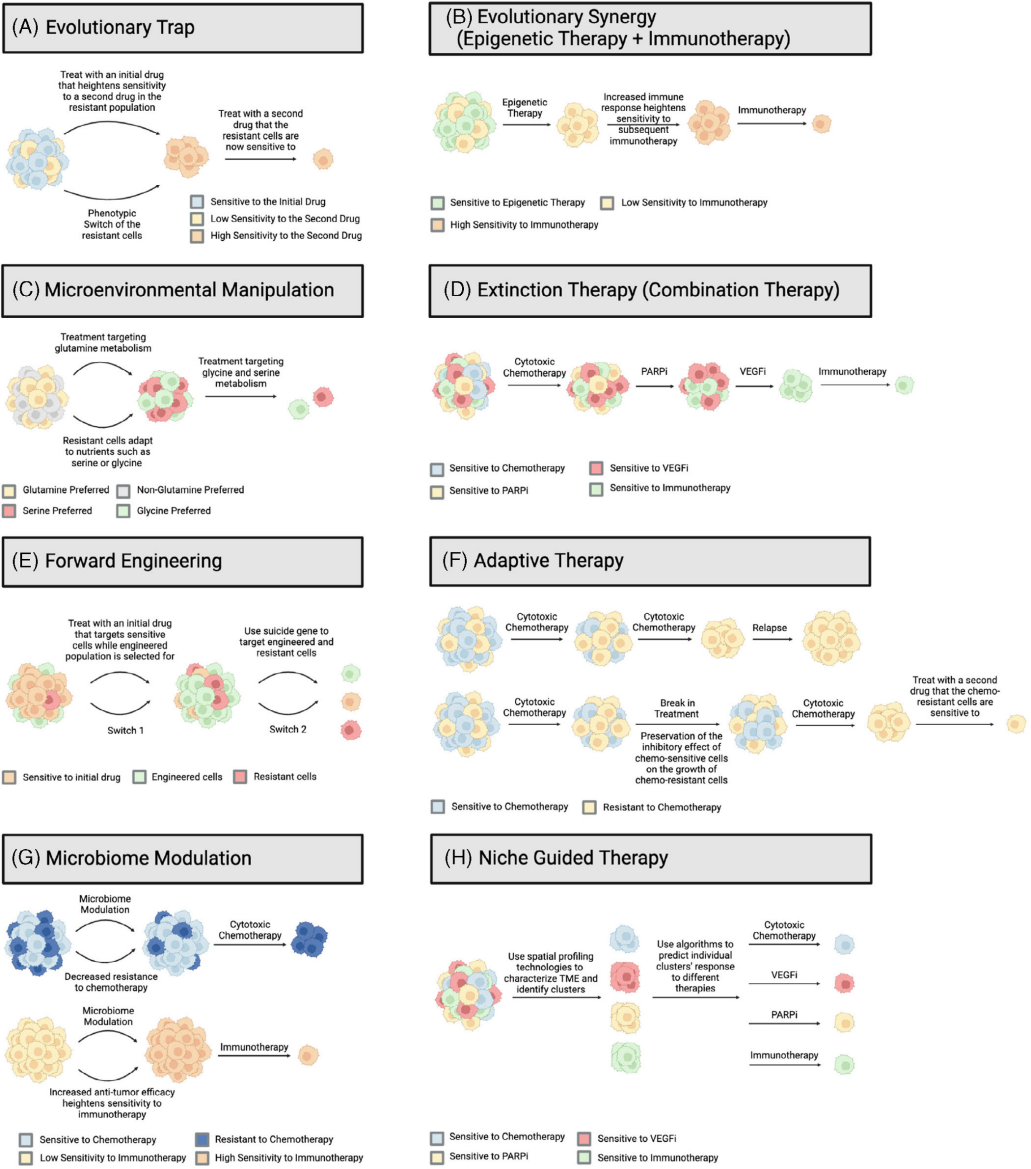
Evolutionarily designed treatments for ovarian cancer considering genetic and non‐genetic tumour heterogeneity. Shading represents cell plasticity. (A) Evolutionary traps can be used when an initial drug is known to induce increased sensitivity to a second drug in the resistant population. This therapy depends on a strong understanding of cell states induced by and vulnerable to treatment. (B) Evolutionary synergy utilises epigenetic therapy, which targets the hypermethylation of cancer cells, leading to reduced suppression of anti‐tumour genes as well as increased immune response that enhances the sensitivity to subsequent immune checkpoint blockade. (C) Microenvironmental manipulation targets the different nutrients that cancer cells depend on for survival based on the understanding that cancer cells adapt to available nutrients in their surrounding environment. (D) Extinction therapy uses a combination of several treatments. Sequential addition of more robust therapies like immunotherapy are used to eliminate cancer cell subpopulations. (E) Forward engineering is a two‐step strategy that uses selection gene drives to eliminate drug‐resistant cancer cells. Initially, sensitive cells are treated with a drug, while engineered cells are selected for survival (switch 1). Once the engineered cells dominate, a suicide gene is then activated to target both engineered and resistant cells (switch 2). (F) In adaptive therapy, pauses in treatment allow for preservation of some sensitive cells to avoid selection of resistant cells. (G) Microbiome modulation utilises the anti‐tumour and pro‐tumour properties of different bacteria to mitigate resistance to chemotherapy while increasing the anti‐tumour efficacy. (H) Niche‐guided therapy combines the use of spatial profiling technologies and mathematical modelling to characterise the tumour microenvironment (TME) and predict individual cluster's response to different therapies.

Evolutionary trap exploits the principles of evolutionary biology where evolved traits that confer adaptability to a given environment may become maladaptive following changes to that environment, with the potential to drive local extinction.^101^ When applying this concept to cancer treatment, two‐body chemotherapeutic regimens can be designed to eradicate cancer cell populations. Understanding this framework requires a discussion of two foundational concepts. Drug‐induced antagonistic pleiotropy (AP) is the idea that genes can exert opposite effects on fitness when exposed to different drugs,^102^ while collateral sensitivity refers to a scenario when acquired resistance to one therapy induces heightened sensitivity to a second therapy.^103^ Therefore, adaptation to one therapy can prime an evolutionary trap, given that a second drug is carefully selected based on AP. Lin et al. demonstrated a drug‐induced AP pathway where resistance to bromodomain inhibition drives sensitivity to BCL‐2 inhibition in acute myeloid leukaemia (AML).^104^ Treatment of AML cells with bromodomain inhibition resulted in a compensatory upregulation of *MYC*, an AP gene that confers sensitivity to BCL‐2 inhibition. Similarly, this method has been successful in treating melanoma. Methotrexate mediates phenotype switching to increased tyrosinase expression, driving sensitivity to a second tyrosinase‐processed antifolate prodrug with melanoma‐specific apoptotic activity.^105^ This framework potentially allows the rational design of evolutionary traps for ovarian cancer by identifying APs and their resultant collateral sensitivities (Figure 4A).

In addition to evolutionary traps, evolutionary synergy achieved through combining epigenetic therapy and immunotherapy presents as alternative strategies for ovarian cancer. Cancer often promotes hypermethylation of promoter‐associated CpG islands, leading to tightly knit chromatin structures and thus silencing of genes that are essential for cellular homeostasis. High levels of H3K9 methyltransferase G9a, a chromatin modifying enzyme, have been shown to correlate with HGSOC and shorter survival.^106^ However, single‐agent epigenetic therapies such as DNA‐methyltransferase inhibitors (DNMTi's) and histone deacetylase inhibitors (HDACi's) have been disappointing in clinical trials for ovarian cancer. Instead, there has been an increasing focus on the potential of epigenetic therapies in combination with other therapies like immunotherapy.^106^ In both murine ovarian cancer model and cell lines, the treatment of DNMTi 5‐azacytidine (AZA) combined with class I HDACi ITF2357 significantly increased the expression of endogenous retroviruses, thereby promoting T‐cell recruitment to the tumour and activating a type I interferon response. When co‐administering with a class II HDACi as well as anti‐PD‐1 ICI, synergy was achieved as the immune response was further potentiated.^106^ The resulting increase in T cells and natural killer cells in the TME bears clinical significance as it attempts to combat the immunologically ‘cold’ nature of ovarian cancer, increasing their susceptibility to immune checkpoint blockade (Figure 4B).

TME manipulation through nutrient deprivation presents as another possibility for managing ovarian cancer. Ovarian tumours exhibit a highly plastic metabolism due to metabolic reprogramming.^107^ This plasticity allows cancer cells to adapt to various environmental conditions while supporting their survival, growth and resistance to therapies. Specifically, there is evidence that some ovarian CSCs favour glycolysis while others may shift towards a state of oxidative phosphorylation (OXPHOS). Interestingly, non‐CSCs also display similar metabolic plasticity, alternating between glycolysis and OXPHOS. This metabolic preference is context dependent, impacted by the TME's conditions (i.e., altered nutrient availability), the need to differentiate and treatment‐induced stress.^107^ As cancer cells adapt to hypoxic and nutrient starved conditions, this plasticity may contribute to disease progression and the development of drug resistance. Glutaminase expression is increased in ovarian cancer, indicating a strong reliance on glutamine metabolism for tumour cell survival.^108^ Compound 968, an allosteric inhibitor of glutaminse 1, significantly inhibited ovarian cancer cell proliferation and increased sensitisation to paclitaxel.^109^ In another preclinical study, Compound 968 in combination with PD‐L1 blockade demonstrated enhanced immune response against ovarian cancer through increased T‐cell infiltration and cytokine production.^110^ Given that ovarian cancer cells display metabolic plasticity, blocking glutamine metabolism may induce cells to adapt to other resources such as serine or glycine (Figure 4C). Identifying additional strategies to starve cancer cells of necessary nutrients might globally suppress tumourigenesis, regardless of individual genetic or non‐genetic variance.

Without the need to identify entirely new treatments, a combination of existing therapies can be utilised to attack different aspects of tumour survival and prevent phenotype switching towards more aggressiveness (Figure 4D). In paediatric sarcoma, most current frontline therapies rely on a combination of maximally tolerated doses of cytotoxic drugs. This mimics the dynamics of a mass extinction, driving cure rates up to 65%−80% for localised disease.^111^ Because tumours rely on multiple pathways for survival and have the capacity to change preference based on external stimuli, combination therapies may promote cell death by minimising viable escape pathways. This multi‐faceted approach may decrease the population of drug persistent or resistant cells while preventing disease recurrence.^112^

Another potential approach that integrates eco‐evolutionary dynamics into ovarian cancer research involves programming tumour evolution with selection gene drives (Figure 4E). Leighow et al. developed a genetic system that manipulates cancer cell populations through two phases.^113^ Initially, a fraction of tumour cells is modified to become gene drive cells upon tumour detection. During the switch 1 phase, these gene drive cells gain a fitness advantage, allowing them to become the dominant population. Once this occurs, the switch 2 phase begins, activating a suicide gene that triggers cell death in those gene drive cells. This system also accounts for potential points of failure, including mutations that may confer resistance to therapy or disrupt the suicide gene's activity. Both in vitro and in vivo results showed that this system was able to target and eliminate cells that would typically develop resistance.^113^ This method demonstrates potential for proactively directing tumour evolution to enhance the efficacy of cancer therapies and prevent relapse.

Due to the significant role of temporal dynamics in the development of drug resistance, dosing schedules for treatments must be considered. Cells within advanced tumours display a continuum of resistance, with some being more susceptible to therapy than others.^88^ Many anti‐cancer drugs are administered following the conventional schedule with increasing but non‐lethal doses over time. This allows a subset of cells who tolerate the non‐lethal dose to prepare for their next exposure, ultimately driving resistance. Conversely, intermittent dosing schedules may help prevent resistance. A drug is initially administered at continuously higher doses to kill the sensitive cells. It is then intermittently paused, during which cells may revert to a more sensitive, treatment‐susceptible state (Figure 4F). Meanwhile, maintenance of some drug‐sensitive populations is crucial due to their role as competitors to drug‐resistant cells.^114^ Without these competitors, drug‐resistant cells may proliferate during the pause, leading to relapse.

Recently, the complex role of the human microbiota has garnered attention in its role in cancer development and therapeutic response. The gut microbiota participates in various molecular pathways to contribute to oncogenesis, including DNA damage and epigenetic alterations, interference with the DDR, abnormal signalling pathways and immune suppression.^115^ In ovarian cancer, gut microbiome dysbiosis leads to elevated levels of pro‐inflammatory cytokine IL‐6, which activates the JAK/STAT3 pathway, promoting cancer progression.^116^ Therefore, modulating the microbiota composition might be able to improve the efficacy of anti‐cancer drugs. Chambers et al. demonstrated that mice treated with antibiotics experienced increased cisplatin resistance and accelerated ovarian cancer growth. Importantly, cecal transplant of mice with healthy microbiomes mitigated the chemotherapy resistance, significantly prolonging their lifespan.^117^ While chemotherapy remains the major cancer treatment, not all patients respond well to it. One possible explanation is due to the individual difference in the gut microbiota composition. In antibiotic‐treated and germ‐free mice, the anti‐tumour efficacy of cyclophosphamide is reduced due to a lack of Th1‐ and Th17‐mediated immune response. Notably, administering the bacteria *Enterococcus* and *Barnesiella* restores the anti‐tumour efficacy by stimulating CD8+, CD4+, Th1 and Th17 cells.^115^ Several approaches to modify the gut microbiome, such as faecal microbiota transplantation, dietary modifications and probiotic supplementation are undergoing active research. Microbiome modulation represents a promising frontier in ovarian cancer therapy, with the potential to mitigate treatment resistance and enhance treatment efficacy (Figure 4G).

Lastly, we propose niche guided therapy that utilises spatial profiling technologies and mathematical algorithms together. Spatial transcriptomic and proteomic analyses allow us to characterise the spatial profiling of TME, which includes the distribution and proportion of immune cells, the activated or suppressed state of immune cells, the distances between immune cells and their nearest functional‐related neighbours, and the direct cell–cell interaction patterns.^118^ Stur et al. analysed HGSOC tumour tissues from poor and excellent responders to chemotherapy. In addition to the differences in tumour composition between the two groups, the spatial interactions between cell clusters played a more influential role in determining chemotherapy responsiveness.^119^ With the advancement of spatial profiling technologies, we propose combining such strategy with mathematical modelling to characterise cluster crosstalk within the TME and predict treatment response (Figure 4H). We have previously shown how mathematical modelling can be used to test patient‐specific PARPi treatment schedules, predict response and optimise treatment accordingly. This adaptive therapy algorithm indicates that adjusting doses is superior to implementing treatment holidays, which can allow PARPi‐resistant cells to persist.^120^

## 5 CONCLUSION

In this review, we have highlighted the current state of ovarian cancer treatment, discussed ongoing research efforts, and made propositions for future directions. Existing treatments have shown modest success in improving patient survival. However, they often fail to account for tumour heterogeneity, tumour evolution, cancer cell plasticity or the TME complexity, leading to treatment failure and relapse. To accommodate these eco‐evolutionary parameters, we have proposed revised treatment strategies, compiled in Figure 4, to prevent disease resistance and recurrence. These suggestions emphasise the necessary role of translational research in bridging the gap between basic research and clinical applications. We believe the essential models and therapies discussed here offer more comprehensive methods for approaching eco‐evolutionary‐based ovarian cancer treatment. Furthermore, while each type of cancer has its unique characteristics and tumour ecosystem, the underlying principles of tumour evolution and resistance are global. The strategies we propose for ovarian cancer can potentially be applied to other tumour types as well to improve the effectiveness of cancer treatments.

## AUTHOR CONTRIBUTIONS

Grace Y.Q. Han, Monica Alexander, Julia Gattozzi, Marilyn Day, Elayna Kirsch, Narges Tafreshi and Mehdi Damaghi wrote the article. Mehdi Damaghi conceptualised the study and developed the idea. All authors reviewed the article and provided final approval of the version submitted for publication.

## ETHICS STATEMENT

This is a review paper and we dont have any animal studies or human or patient related studies in this work.
